# Seventh Annual Meeting of the Tri-State Medical Society of Alabama, Georgia and Tennessee

**Published:** 1895-11

**Authors:** 


### SEVENTH ANNUAL MEETING OF THE TRI-STATE MEDICAL SOCIETY OF ALABAMA, GEORGIA AND TENNESSEE.

CHATTANOOGA, TENN., October 10, 1895.

Called to order by the President, R. M. Cunningham, who presided during the whole session.

After hearing reports of the Secretary and Treasurer, and the transaction of some miscellaneous business, the President delivered his address, entitled "Tuberculosis," based upon observations of that disease in the Alabama penitentiary, notably at Pratt City, Alabama.

For a period of twenty years, ending October, 1889, the mortality from tuberculosis was 19.33 per cent. of the total mortality from disease.

He concluded from statistics from 1883 to 1895, that, out of an equal number of white and negro convicts, there were seven deaths among the negroes to one among the whites from tuberculosis. The figures demonstrate the wonderful racial predisposition of the negro race to tuberculosis compared to the white, under precisely the same environment, which he explains as follows:

1. The negro has acquired since his emancipation a predisposition to tuberculosis, which for the present and succeeding generations is hereditary.

2. The greater liability to diseases which produce a local predisposition to tuberculosis, notably bronchial and intestinal catarrh and pleurisy.

3. He is physically, mentally and morally inferior to the white man; therefore,

4. He is more liable to contract disease, particularly tuberculosis and thoracic diseases (local and from infection) generally.

5. His changed social, religious, political and industrial relations, involving as a rule a change from the segregate to the aggregate—from country to town, and from farm to public works.

6. His disregard for all rules of sanitation.

The greatest mortality was between twenty and thirty years of age. In an analysis of seventy-one cases, thirty-five had been in prison less than one year. Many were diseased when received. The figures demonstrated an endemic from April to September, 1895.

There are two prisons at Pratt City, a mile apart, number of inmates about equal, under the same management and hygiene. At No. 1, forty-five deaths; No. 2, twenty-six. Difference in favor of No. 2, 19, explained as follows: From tubercular peritonitis there died at No. 1, twenty; at No. 2, one; difference nineteen, exactly the difference in mortality from tuberculosis. This difference was due to an epidemic of diarrhea at No. 1, proving that intestinal catarrh predisposes to tuberculosis, especially the pritoneal form. He always found an epidemic of diarrhea to be followed by a large number of cases of tubercular peritonitis.

The causes of tuberculosis were dividid into the essential and predisposing. 1st. The essential: the tubercle bacillus; 2d. The predisposing: (*a*) an inherited constitutional predisposition, (*b*) an acquired constitutional or local predisposition. The predisposing *never alone* producing, and the essential rarely, the two acting together producing the disease.

He classified tuberculosis into: 1st, acute general tubercuolosis; 2d, chronic general tuberculosis; 3d, acute local tuberculosis; 4th, chronic local tuberculosis.

Primarily it is the result of hetero-inoculation, and, as a rule, almost without exception, is a local infection producing chronic local tuberculosis, the general and acute forms being the result of auto-infection, the type, site and extent of the disease depending on the constitutional or local predisposition and the number and manner of the introduction of the tubercle bacilli.

In general acute miliary tuberculosis, the lungs, peritoneum, spleen and sometimes the liver, rarely the kidneys, were involved, the patients dying before the caseous stage of the process was reached. These cases most frequently followed pleurisy, notably after aspiration with a large amount of fluid withdrawn, and from tubercular glands.

In the chronic general form the process involves the same organs, but the infection is not so general and the caseous stage is often reached and local inflammatory lesions generally found.

In both there may be an active tubercular inflammation developed in some organs, rapidly terminating the disease. The bacilli were distributed by the circulation.

In the local forms the acute as a rule assumes the form of an acute inflammation. The inflammatory lesions, however, may be slight, the *acuteness* and *severity* depending upon the intensity of the local infection and tubercular process. These cases usually result from a local auto-infection from a chronic tuberculosis which may not have been detected or even suspected. He has found tuberculosis of bone in the negro rare. The acute form very rare. The bone most often affected he has found to be the sternum.

G. MANNING ELLIS read a paper, "Pseudo-Hypertrophic Muscular Paralysis," and presented a patient giving the characteristic history. The boy was late in attempting to walk. The muscles seemed to be large and well developed. The gait was oscillating. The child, while seeming to be well developed and otherwise healthy, showed an increase in the difficulty of locomotion. The characteristic symptoms were shown: The peculiar gait, the lordosis, the manner of rising by placing the hands on the knees and then on the thighs which is almost pathognomonic, the absence of the tendon reflex and the diminution in the size of the muscles of the legs which comes after the enlargement. The upper extremities show the enlarged infraspinatus and the decrease in size of the latissimus dorsi, producing an absence of the axillary fold.

J. B. MURFREE read a paper on "The Placenta; When and How Delivered." He thought that as soon as the child was born Credé's method should be employed. If the placenta does not come away in twenty minutes, gentle traction should be made on the cord. Undue force should not be exerted.

If this does not succeed, and especially if the placenta presents at the os centrally, the hand should be introduced and the edge of the placenta freed from its attachments. If the placenta was delivered as soon as the child was born, it would leave the mouths of the vessels open. Exactly when to remove it cannot be definitely stated in every case. Most practitioners wait too long.

R. R. KIME took issue with the author in regard to introducing the hand into the uterus, as that would greatly add to danger of infection. It is rarely necessary to introduce the hand. By wrapping the cord around the fingers of the right hand and pressing the posterior lip of the cervix backward, the placenta will slip out. He protested against ergot, which contracted the os and prevented the placenta from coming out.

W. G. BOGART agreed with the writer in the main. He proceeds to deliver placenta as soon as he ties the cord, gives the child to the nurse, and prepares his hands, which takes about twenty minutes. He grasps the fundus and squeezes the placenta out, if possible. If not successful, he introduces two fingers into the uterus and makes a rotary motion, with the other hand still on the fundus. Never makes traction on the cord, which he deems dangerous. Ergot should not be given to expel the placenta.

An important point was to get all the placenta away. Has no fear of putting the hand into the uterine cavity if it was thoroughly cleansed, nor does he deem it necessary for this reason to use an intrauterine douche.

PRESTON SCOTT thought that we were all too hasty to get rid of the placenta. Time should be given for tonic contraction. Gentle traction should be made on the twisted body of the placenta. Had excluded ergot from his practice entirely. He used the hot water douche when the delivery was slow, to promote contraction.

J. P. STEWART waits longer than twenty minutes—an hour if there is no hemorrhage. If the placenta is in the vagina, he delivers at once, as it acts as a foreign body, causing contraction and tearing the membranes.

J. B. COWAN thought there was a happy medium between twenty minutes and an hour. He assists nature during the pains. If necessary he puts his hand into the uterus. Post-partum hemorrhage can be prevented by steady pressure over the fundus, kept up for an hour if necessary. He never pulls the cord.

WILLIS F. WESTMORELAND presented: "Report of Cases: (*a*) Tracheotomy for Foreign Bodies; (*b*) Cystotomy for Stone."

He reported twenty-seven successful cases of tracheotomy for foreign bodies.

In all the tracheotomy was made by cutting the first ring and enlarging downward, as necessary. Operations were made as early as possible. The external incision was made large. Muscles were separated, and the fascia divided; vessels were ligated so that the forceps were in the road.

If necessary, the isthmus of the thyroid was divided and ligated. No tenaculum was used to steady the trachea. Instead of trachea forceps, the wound was held open with silk thread introduced with a needle having no cutting edge. The foreign body was not probed for, but if it was not coughed out, the wound was left open, in one case (cocklebur) for three days. In closing the wound, the tissue under the mucous membrane is brought together with silk, using a needle with no cutting edge and the Halsted or mattress suture. In the same way layer after lay is brought together. The child is up in twenty-four hours.

In cystotomy, he avoids rectal dilatation. To distend the bladder he uses hydrostatic pressure after the opening in the abdomen, having the water at a height of two feet. This causes the bladder to bulge up through the abdominal opening. No tenaculum is used to steady the bladder, but it is held with a pair of forceps on each side of the proposed opening, which is made as high as possible. After removal of the stone, the wound in the bladder is closed with the Halsted suture and the bladder is flushed out. The closure is tested by raising the water three feet. He reported a case where a stone had been removed weighing six ounces. The bladder had been injured five years previously.

G. A. BAXTER indorsed the position of the writer. He was attached to the medio-lateral operation. Stone is rare in this section. Not a dozen cases have been operated on in the last fifteen years. The disease was more frequent in Middle Tennessee. He advocated drainage in the supra-pubic operation.

J. A. GOGGANS related a case in which the larynx at the site of the thyroid cartilage was almost occluded by a new growth, so that he had to operate rapidly to save the life of the patient. Throwing the head back increased the difficulty of breathing, so that it had to be thrown forward and the larynx steadied with a tenaculum, which, he thought, did no harm.

The supra-pubic was the operation *par excellence* for stone. He was somewhat prejudiced against the perineal operations, and related a case sent to him where a large stone had been removed through the perineum, and a large fistulous opening left between the bladder and rectum. He had attempted to repair this by silk-worm gut, operating through the rectum. At the third operation had divided the sphincter and muscle in front down to the fistulous tract, and had almost closed the opening.

W. E. B. DAVIS stated that he was glad that both operations were fairly considered. The results of the older surgeons who performed the low operations were good. He found that statistics of the supra-pubic operation were bad because unfavorable cases were selected for this operation. A distinguished surgeon had so operated in a bad case to save his statistics for the low operation. This shows how statistics can be doctored. He thought it well to leave the wound open for drainage, as there is always disease, and a new stone may form in a few weeks.

R. J. TRIPPE said that his experience with the supra-pubic operation was limited to a few cases operated on for disease, and advocated leaving the wound open for drainage. As a guide he used a sound and did not fill the bladder, but always washed it out before the operation. In tracheotomy he used long scissors forceps. These saved time to tie vessels; saved an assistant, as they held the wound open. He held the trachea open with retractors made of hairpins fastened to tape.

In closing the discussion, Dr. Westmoreland said that he used no instrument to hold the trachea open, as that took up some room, while the silk did not. Time is of importance, but we always have two minutes after a child quits breathing, as resuscitation can be effected after that. He advocated cutting high in supra-pubic cystotomy, as the bladder is near the surface, being quite deep at the pubis. The tenaculum was avoided, as urine might be voided through the small opening. The buried silver suture was used, owing to the fact that it took several weeks for the muscular tissue to unite, and no other suture would hold that long. Without this suture, according to the statistics of Gregg-Smith, twenty per cent. of cases of abdominal section had hernia.

In the high operation it would make no difference if the peritoneum were cut, as the bladder was filled with an antiseptic solution. The wound in these cases was not left open for drainage, as they were not cases of disease.

J. A. Goggans read a paper, entitled: "Early Diagnosis and Vaginal Hysterectomy in Cancer of the Uterus," dwelling on the importance of prophylaxis, early diagnosis and early operative treatment by vaginal hysterectomy.

A. R. Robinson read a paper entitled: "The Treatment of Malignant Cutaneous Epitheliomata" (Cancers). In cases where the diagnosis is not positive iodide and mercury should be used. The elements in these cases extend much farther than is generally supposed. When they are cut out pathological cells are left and have so-called recurrence, which is really a reappearance, when the wound has been treated antiseptically. It should be allowed to suppurate, as the toxine of the pus is more destructive of the epithelioma cells than the erysipelas toxine. He opposed cutting, and advocated caustics. The toxines had cured no cases; some may have been benefited. The caustic should destroy the tissue completely and quickly. The milder caustics, as nitrate of silver, should not be used.

Caustic potash quickly liquefies the tissues as much as would be removed with the knife. Beyond this, there is an inflammatory exudation which destroys the pathological elements. The cancer cells may extend deeper than could be reached with the knife, and here the potash is preferable. There is less deformity with this than with the knife, more than with arsenious acid.

Chloride of zinc acts more slowly, suitable only in certain locations, as near the eye, and in the papillary form previous to the use of arsenious acid. Pain may be avoided by mixing it in a 20 per cent. solution of cocaine.

Arsenious acid has more of an elective effect than the above agent, and should, as a rule, be used weaker than Marsden's paste (2 to 1 of gum acacia). It may be used 6 to 4, or equal parts. This will not attack normal tissue in twenty hours. It should then be removed, and if there is not sufficient necrosis, apply again immediately sixteen to eighteen hours. The cases should be watched for a year or two, as there may be cells not destroyed. Arsenious acid may be applied on the lip, if care is taken to prevent getting it in the mouth, but on the nose it is especially valuable on account of deformity.

These papers were discussed together.

R. R. KIME said that it was unfortunate that cancer of the uterus was not diagnosed early. The symptoms were not well defined, even when the disease was so far advanced that an operation could not be performed. Cases from 35 to 50 should be examined, if any suspicion of cancer. If the general practitioner is in doubt he should refer the case to the gynecologist. If confined to the cervix this may be removed, followed by caustics. The fatal results of amputation of the cervix are so much less than vaginal hysterectomy.

W. E. B. DAVIS thought that physicians overlooked these cases, as they developed so insidiously. Every case of diseased cervix should be treated and cured. He advised removal of the uterus and ovaries for cancer.

(*To be concluded.*)

